# Modular Optimization of Heterologous Pathways for De Novo Synthesis of (2S)-Naringenin in *Escherichia coli*

**DOI:** 10.1371/journal.pone.0101492

**Published:** 2014-07-02

**Authors:** Junjun Wu, Tiantian Zhou, Guocheng Du, Jingwen Zhou, Jian Chen

**Affiliations:** 1 School of Biotechnology, Jiangnan University, Wuxi, Jiangsu, China; 2 Synergetic Innovation Center of Food Safety and Nutrition, Wuxi, Jiangsu, China; University of Houston, United States of America

## Abstract

Due to increasing concerns about food safety and environmental issues, bio-based production of flavonoids from safe, inexpensive, and renewable substrates is increasingly attracting attention. Here, the complete biosynthetic pathway, consisting of 3-deoxy-D-arabinoheptulosonate 7-phosphate synthase (DAHPS), chorismate mutase/prephenate dehydrogenase (CM/PDH), tyrosine ammonia lyase (TAL), 4-coumarate:CoA ligase (4CL), chalcone synthase (CHS), chalcone isomerase (CHI), malonate synthetase, and malonate carrier protein, was constructed using pre-made modules to overproduce (2S)-naringenin from D-glucose. Modular pathway engineering strategies were applied to the production of the flavonoid precursor (2S)-naringenin from L-tyrosine to investigate the metabolic space for efficient conversion. Modular expression was combinatorially tuned by modifying plasmid gene copy numbers and promoter strengths to identify an optimally balanced pathway. Furthermore, a new modular pathway from D-glucose to L-tyrosine was assembled and re-optimized with the identified optimal modules to enable de novo synthesis of (2S)-naringenin. Once this metabolic balance was achieved, the optimum strain was capable of producing 100.64 mg/L (2S)-naringenin directly from D-glucose, which is the highest production titer from D-glucose in *Escherichia coli*. The fermentation system described here paves the way for the development of an economical process for microbial production of flavonoids.

## Introduction

(2S)-Naringenin, a member of the flavonoid family, possesses a broad range of pharmaceutical indications due to its biochemical properties, which include antioxidative, anticancer, and anti-inflammatory activities [1], [2]. Notably, (2S)-naringenin is the starting point for the synthesis of a variety of other flavonoid molecules. Over 8000 different chemical structures can be created through the combined actions of functionalizing enzymes [3]. However, (2S)-naringenin is still chiefly obtained by extraction from plants, which is tedious and inefficient and requires consumption of substantial natural resources. Hence, biological synthesis has emerged as a highly promising alternative to the traditional extraction method for a variety of chemical compounds as it may readily be scaled up for commercial production, utilizes environmentally friendly feedstocks, and has low waste emission [4], [5].

**Figure 1:**
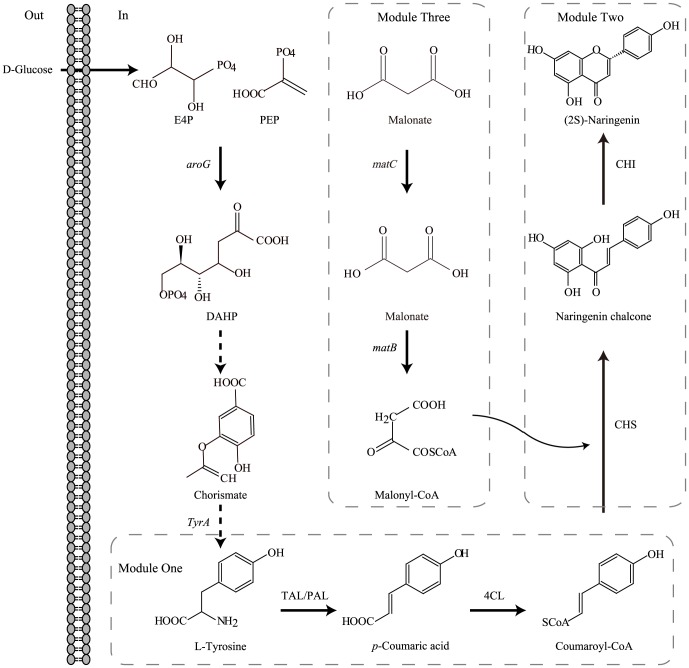
Modular optimization of heterologous pathways for de novo synthesis of (2S)-naringenin. Schematics of the three modules: module one (TAL, 4CL), module two (CHS, CHI), and module three (*matB*, *matC*). *aroG*: the gene encoding 3-deoxy-D-arabinoheptulosonate-7-phosphate (DAHP) synthase. *tyrA*: the gene encoding chorismate mutase/prephenate dehydrogenase (CM/PDH). TAL: tyrosine ammonia lyase; 4CL: 4-coumarate:CoA ligase; CHS: chalcone synthase; CHI: chalcone isomerase. *matB*: the gene encoding *R. trifolii* malonate synthetase; *matC*: the gene encoding *R. trifolii* malonate carrier protein.

In plants, (2S)-naringenin is synthesized via the phenylpropanoid pathway, which is a ubiquitous and well-described plant secondary metabolite pathway [6]. (2S)-Naringenin biosynthesis begins with the enzymatic conversion of L-tyrosine by tyrosine ammonia lyase (TAL) to produce *p*-coumaric acid, which is then converted into its corresponding coenzyme A ester, coumaroyl-CoA, through 4-coumarate:CoA ligase (4CL) (Fig. 1). This compound is subsequently condensed with three malonyl-CoA units by chalcone synthase (CHS), and the resulting (2S)-naringenin chalcone is converted to (2S)-naringenin by the action of chalcone isomerase (CHI) [7].

Although significant progress has been made recently in improving strain titers and yields [8], [9], [10], the established protocols rely heavily on a two-step culture process with phenylpropanoid acid precursors supplemented [11], which is expensive and commercially unfavorable in large-scale fermentation processes. Previous studies have demonstrated the feasibility of de novo production of (2S)-naringenin [11] by optimizing individual pathway components until the desired performance is achieved. However, modifications of individual pathways may not be additive as precursor flux improvement may not be accommodated by downstream pathways. Indeed, some bottlenecks are not revealed until others are relieved. These may result in the accumulation of intermediate metabolites and suboptimal titers [4], [12]. Therefore, cooperative regulation of the overall pathways should generate better results [13].

To achieve direct (2S)-naringenin production from D-glucose, it has become clear from previous studies that efficient conversion of L-tyrosine to (2S)-naringenin is the limiting factor [11]. To investigate the metabolic space for efficient conversion of L-tyrosine to (2S)-naringenin, modular pathway engineering strategies [12] were applied in this study. Modular expression was combinatorially tuned by modifying plasmid gene copy numbers and promoter strengths to identify an optimally balanced pathway. Furthermore, a new modular pathway from D-glucose to L-tyrosine was assembled and re-optimized with the identified optimal modules to enable de novo synthesis of (2S)-naringenin. The optimum strain was capable of producing 100.64 mg/L (2S)-naringenin from D-glucose, which is the highest production titer from D-glucose in *Escherichia coli*.

## Materials and Methods

### *E. coli* strains, plasmids and general techniques

Luria broth (LB) and MOPS minimal medium [14] supplemented with 5 g/L D-glucose and an additional 4 g/L NH_4_Cl were used. Various combinations of ampicillin (100 µg/mL), kanamycin (40 µg/mL), chloramphenicol (20 µg/mL), and streptomycin (40 µg/mL) were added to cultures of plasmid-bearing *E. coli* strains. The primers and plasmids used in this study are given in Tables 1 and 2, respectively. The compatible vectors pETDuet-1, pCDFDuet-1, pRSFDuet-1, pACYCDuet-1, and pCOLADue-1 were purchased from Novagen (Darmstadt, Germany). All restriction enzymes and DNA ligase were also purchased from Novagen (Darmstadt, Germany). Codon-optimized enzymes TAL (GenBank ID: KF765779) from *Rhodotorula glutinis*, 4CL (GenBank ID: KF765780) from *Petroselinum crispum*, CHS (GenBank ID: KF765781) from *Petunia X hybrida*, CHI (GenBank ID: KF765782) from *Medicago sativa*, and *matB* (GenBank ID: KF765783) and *matC* (GenBank ID: KF765784) from *Rhizobium trifolii* for *E. coli* expression were synthesized by GeneScript (Nanjing, China). The accession numbers for unmodified genes of TAL, 4CL, CHS, CHI, *matB*, and *matC* are DQ013364, X13325, AF233638, M91079, AAC83455, and AAC83457, respectively. Gene sequences of these modified genes are provided in the Supporting Information S1. *E. coli* JM109 was used for plasmid propagation and BL21 (DE3) was used for flavonoid production. Polymerase chain reaction (PCR) was performed using *pfu* turbo polymerase (Merck, Darmstadt, Germany).

**Table 1:** Nucleotide sequences of primers.

Oligonucleotides	Sequences, 5′-3′ a	
Pf_Ttrc (*Fse*I)	**GGCCGGCC** CCGACATCATAAC	
Pr_Trc (*Eco*NI)	**CCTGCATTAGG** CAACAGATAAAACGAAAGGCC	
Pf_TAL (*Nco*I)	CATG**CCATGG**CGCCGCGCCCGACTTCTC	
Pr_TAL (*Eco*RI)	CCG**GAATTC**TTATGCCAGCATCTTCAGCAGAACGTTGTTGAT	
Pf_4CL (*Nco*I)	CATG**CCATGG**GTGACTGCGTTGCCCCG	
Pr_4CL (*Hin*dIII)	C**AAGCTT**TTACTTCGGCAGGTCGCCGCTC	
Pf_Ptrc4CL (*Eco*RI)	CCG**GAATTC**CCGACATCATAACGGTTCTGG	
Pf_CHS (*Nco*I)	CATG**CCATGG**TTACGGTGGAAGAATAC	
Pr_CHS (*Eco*RI)	CCG**GAATTC**TTAGGTAGCCACACTATGCAG	
Pf_CHI (*Nco*I)	CATG**CCATGG**CGCAGCAAGCATTACGG	
Pr_CHI (*Hin*dIII)	CCC**AAGCTT**ACCGATTTTAAAGGCA	
Pf_PtrcCHI (*Eco*RI)	CCG**GAATTC**CCGACATCATAACGGTTCTGG	
Pf_matB (*Nco*I)	CATG**CCATGG**GCATGAGCAACCACCTGTTTGATG	
Pr_matB(*Hin*dIII)		CCC**AAGCTT**TCAGGTGCGGGTGTACAGGT
Pf_PtrcmatB(*Eco*RI)		CCG**GAATTC**CCGACATCATAACGGTTCTGG
Pf_matC (*Nco*I)		CATG**CCATGG**GCATCGAACTGCTGAGTATTG
Pr_matC (*Eco*RI)		CCG**GAATTC**TTAGACCAGACCCGGCACAACG
Pf_aroG(*Nco*I)		CATG**CCATGG**CAATGAATTATCAGAACGACGATTTACGC
Pr_aroG(*Hin*dII)		CCC**AAGCTT**TTACCCGCGACGCGCTTTTAC
Pf_tyrA(*Nde*I)		GGGAATTC**CATATG**GTTGCTGAATTGACCG
Pr_tyrA(*Bln*I)		**CCTAGG** TTACTGGCGATTGTCATTCG
Pf_tyrA^fbr^(53)		CGCGAGGCATCTATTTTGGCCTCGCGTC
Pr_tyrA^fbr^(53)		GACGCGAGGCCAAAATAGATGCCTCGCG
Pf_tyrA^fbr^(354)		GTTCGGCGATTACGTACAGCGTTTTCAGAG
Pr_tyrA^fbr^(354)		CTCTGAAAACGCTGTACGTAATCGCCGAAC
Pf_aroG^fbr^(146)		CAGGTGAGTTTCTCAATATGATCACCCC
Pr_aroG^fbr^(146)		GGGGTGATCATATTGAGAAACTCACCTG
Pf_Kan^FRT^(*pfo*I)		**TCCGGGA** GTGTAGGCTGGAGCTGCTTC
Pr_Kan^FRT^(*Eco*NI)		**CCTAATGCAGG** CTGTCAAACATGAGAATTAATT

a : Bold and underlined letters are restriction enzyme cut sites.

**Table 2:** Plasmids used in this study.

Plasmids	Description	Source or reference
pCDFDuet-1	Double *T7* promoters, CDF ori, Sm^R^	Novagen
pETDuet-1	Double *T7* promoters, pBR322 ori, Amp^R^	Novagen
pACYCDuet-1	Double *T7* promoters, P15A ori, Cm^R^	Novagen
pRSFDuet-1	Double *T7* promoters, RSF ori, Kn^R^	Novagen
pCOLADuet-1	Double *T7* promoters, ColA ori, Kn^R^	Novagen
pET-Trc	*T7* promoter was replaced by *Trc* promoter	This study
pCDF-TAL-4CL	pCDFDuet-1 carrying TAL and 4CL	This study
pCDF-Trc	*T7* promoter was replaced by *Trc* promoter	This study
pCDF-Trc-TAL	pCDFDuet-1 carrying TAL under *Trc* promoter	This study
pCDF-Trc-TAL-Trc-4CL	*T7* promoter was replaced by *Trc* promoter	This study
pACYC-TAL-4CL	pACYCDuet-1 carrying TAL and 4CL	This study
pACYC-Trc	*T7* promoter was replaced by *Trc* promoter	This study
pACYC-Trc-TAL	pACYCDuet-1 carrying TAL under Trc promoter	This study
pACYC-Trc-TAL-Trc-4CL	*T7* promoter was replaced by *Trc* promoter	This study
pACYC-matC-matB	pACYCDuet-1 carrying *matB* and *matC*	[9]
pACYC-Trc-matC	pACYCDuet-1 carrying *matC* under Trc promoter	This study
pACYC-Trc-matC-Trc-matB	*T7* promoter was replaced by *Trc* promoter	This study
pCDF-Trc-matC	pCDFDuet-1 carrying *matC* under Trc promoter	This study
pCDF-matC-matB	pCDFDuet-1 carrying *matB* and *matC*	This study
pCDF-Trc-matC-Trc-matB	*T7* promoter was replaced by *Trc* promoter	This study
pET-Trc-matC	pETDuet-1 carrying *matC* under *Trc* promoter	This study
pET-matC-matB	pETDuet-1 carrying *matB* and *matC*	This study
pET-Trc-matC-Trc-matB	*T7* promoter was replaced by *Trc* promoter	This study
pCDF-CHS-CHI	pCDFDuet-1 carrying CHS and CHI	This study
pCDF-Trc-CHS-Trc-CHI	*T7* promoter was replaced by *Trc* promoter	This study
pET-CHS-CHI	pETDuet-1 carrying CHS and CHI	This study
pET-Trc-CHS-Trc-CHI	*T7* promoter was replaced by *Trc* promoter	This study
T-aroG(WT)	T-vector pMD^TM^19 (Simple) carrying *aroG* (WT)	This study
T-tyrA(WT)	T-vector pMD^TM^19 (Simple) carrying *tyrA* (WT)	This study
T-tyrA^fbr^	T-vector pMD^TM^19 (Simple) carrying *tyrA* ^fbr^	This study
T-aroG^fbr^	T-vector pMD^TM^19 (Simple) carrying *aroG* ^fbr^	This study
pCDF-tyrA^fbr^-aroG^fbr^	pCDFDuet-1 carrying *tyrA* ^fbr^ and *aroG* ^fbr^	This study
pRSF-tyrA^fbr^-aroG^fbr^	pRSFDuet-1 carrying *tyrA* ^fbr^ and *aroG* ^fbr^	This study
pCOLA-tyrA^fbr^-aroG^fbr^	pCOLADuet-1 carrying *tyrA* ^fbr^ and *aroG* ^fbr^	This study
pCDF-Kan^FRT^-tyrA^fbr^aroG^fbr^	pCDFDuet-1 carrying *Kan* ^FRT^-*tyrA* ^fbr^-*aroG* ^fbr^ cassette	This study

### Culture conditions

For flavonoid production from L-tyrosine, strains were first cultured in 25 mL of MOPS medium at 37°C with 220 rpm orbital shaking. After an OD_600_ of 1.65 had been reached, an additional 25 mL of fresh MOPS medium, a final aliquot of isopropyl-β-D-thiogalactopyranoside (IPTG) (taking the concentration to 1 mM), and a final aliquot of L-tyrosine (taking the concentration to 3 mM) were added. Cultures were subsequently conducted at 30°C for (2S)-naringenin production. (2S)-Naringenin concentrations were measured after a total fermentation time of 48 h. For malonyl-CoA availability experiments, 1 g/L of sodium malonate dibasic (Sigma) was added twice, resulting in a total concentration of 2 g/L.

For flavonoid production from D-glucose, strains were first cultured in 25 mL of MOPS medium until an OD_600_ of 1.65 was reached, after which an additional 25 mL of fresh MOPS medium and a final aliquot of IPTG (taking the concentration to 1 mM) were added. Cultures were subsequently conducted at 30°C for (2S)-naringenin production. For malonyl-CoA availability experiments, sodium malonate dibasic (Sigma) was added at a concentrations of 2 g/L (1 g/L added twice).

### Flavonoid analysis and quantification

To analyze (2S)-naringenin and *p*-coumaric acid production, *E. coli* cells were separated through centrifugation (5000 g, 15 minutes, 4°C). To quantify levels of (2S)-naringenin, 1 mL of supernatant was extracted with an equal volume of ethyl acetate (EMD Chemicals, Darmstadt, Germany). After vortexing and centrifugation (5000 g, 15 minutes, 4°C), the top organic layer was separated and evaporated to dryness, and the remaining residue was resolubilized with 1 mL of methanol (EMD Chemicals, Darmstadt, Germany). Samples were analyzed by high-performance liquid chromatography (HPLC), using an Agilent 1100 series instrument and a reverse-phase Gemini NX-C18 column (5×110 mm) maintained at 25°C. (2S)-Naringenin was separated by elution with an acetonitrile/water gradient at a flow rate of 1.0 mL/min under the following conditions: 10% to 40% acetonitrile (vol/vol) for 10 min, 40% acetonitrile (vol/vol) for 5 min, 40% to 10% acetonitrile (vol/vol) for 2 min. The retention times under these conditions for standard authentic samples of (2S)-naringenin (Sigma-Aldrich, W530098-SAMPLE) and *p*-coumaric acid (Sigma-Aldrich, 55823-50 mg) were 14.085 and 10.481 min, respectively. The recombinant product was detected by monitoring absorbance at 280 nm.

The compound was identified by the area of major mass spectra signals ([M–H]^−^) accumulated on a liquid chromatography-mass spectrophotometer (LC-MS) (Shimadzu, Kyoto, Japan) equipped with an electrospray ionization (ESI) source. HPLC separations were performed using an Agilent Zorbax Extend-C18 column (4.6 mm×150 mm, 5 µm) under the gradient elution mode at a flow rate of 0.7 mL/min. Mobile phases A and B were water and acetonitrile, respectively, both containing 0.1% formic acid. Gradient elution was conducted as follows: 0–20 min for 5–60% B with a linear gradient, followed by 20–30 min of 100% B [15]. The MS/MS system was operated in electrospray ionization (ESI) mode. Typical operating parameters were as follows: detector voltage, 1.60 Kv; nebulizing gas (N_2_) flow, 1.5 L/min; drying gas (N_2_) flow, 200 kPa; ion accumulation time, 30 ms; scan range m/z, 100–1000 for MS^1^, 100–500 for MS^2^. The instruments used for quantitative measurements were operated in the selected ion monitoring (SIM) mode. Data-dependent tandem mass spectrometry experiments were controlled using Shimadzu Composition Formula Predictor software. The negative ion value by LC-MS of the authentic (2S)-naringenin compound was 271.0591 [M-H]^−^.

### Construction of pCDF-Kan^FRT^-tyrA^fbr^aroG^fbr^ plasmid

Chromosomal DNA from *E. coli* K12 was prepared by using an Ezup Column Bacteria Genomic DNA Purification Kit (Sangon Biotech, Shanghai, China). Primers Pf_aroG(*Nco*I) and Pr_aroG(*Hin*dIII), Pf_tyrA(*Nde*I) and Pr_tyrA(*Bln*I) were used to clone the wild-type (WT) *aroG* and *tyrA* with *pfu* turbo polymerase. These PCR products were then ligated into T-vector pMD^TM^19 (Simple) (Takara, Dalian, China), which resulted in plasmids T-aroG(WT) and T-tyrA(WT). Two amino acid substitutions were performed to obtain feedback-inhibition-resistant (fbr) *tyrA* gene (*tyrA* ^fbr^): Met-53-Ile in the chorismate mutase domain and Ala-354-Val in the prephenate dehydrogenase domain [16]. An Asp-146-Asn substitution was performed to obtain feedback-inhibition-resistant (fbr) *aroG* gene (*aroG* ^fbr^) [17]. The QuikChange II XL Site-Directed Mutagenesis Kit (Agilent Technologies, Santa Clara, CA) and primers Pf_tyrA^fbr^(53) and Pr_tyrA^fbr^(53), Pf_tyrA^fbr^(354) and Pr_tyrA^fbr^(354), Pf_aroG^fbr^(146) and Pr_aroG^fbr^(146) were used to perform these substitutions. The resulting plasmids were named T-tyrA^fbr^ and T-aroG^fbr^, respectively.

pCDF-tyrA^fbr^ was constructed by cloning *tyrA* ^fbr^ from T-tyrA^fbr^ into *Nde*I/*Bln*I sites of pCDFDuet-1. pCDF-tyrA^fbr^-aroG^fbr^ was constructed by cloning *aroG* ^fbr^ from T-aroG^fbr^ into *Nco*I/*Hin*dIII sites of pCDF-tyrA^fbr^. To construct pCDF-Kan^FRT^-tyrA^fbr^-aroG^fbr^, primers Pf_Kan^FRT^(*pfo*I) and Pr_Kan^FRT^(*Eco*NI) were used to amplify an FRT-flanked kanamycin resistance gene (*Kan*) (Kan^FRT^) on the plasmid pKD13 [18]. After digestion with *pfo*I and *Eco*NI, this product was ligated to *pfo*I/*Eco*NI sites of pCDF-tyrA^fbr^-aroG^fbr^.

### Chromosomal integration of the tyrA^fbr^-aroG^fbr^ cassette

The *Kan* ^FRT^-*tyrA* ^fbr^-*aroG* ^fbr^ cassette was integrated into the *lacZ* locus of *E. coli* BL21 using a lambda-red recombination-based method [18]. Briefly, *Kan* ^FRT^-*tyrA* ^fbr^-*aroG* ^fbr^ was amplified from pCDF-Kan^FRT^-tyrA^fbr^aroG^fbr^ with primers Pf_Kan^FRT^-tyrA^fbr^-aroG^fbr^ (CCAGGCTTTACACTTTATGCTTCCGGCTCGTATGTTGTGTGAAATTGTGAGCGGATAACAATTTCACACAGGAAACAGCTGTGTAGGCTGGAGCTGCTTCG) and Pr_Kan^FRT^-tyrA^fbr^-aroG^fbr^ (CAAAAGTTTGTGTTTTTTAAATAGTACATAATGGATTTCCTTACGCGAAATACGGGCAGACATGGCCTGCCCGGTTATTACCTAGGTTACTGGCGATTGTCATT). Both primers incorporated 80 bp of homology with the ends of the *lacZ* gene to facilitate integration into the proper locus. Following transformation of the cassette into *E. coli* BL21, colonies were verified by colony PCR and sequencing. Excision of FRT-flanked *kan* from the resulting strains *E. coli lacZ*::*Kan* ^FRT^ *-tyrA* ^fbr^ *-aroG* ^fbr^ was mediated by transformation with FLP recombinase-expressing pCP20 as described in the literature [18].

### Heterologous pathway construction and assembly

All constructed plasmids were verified by both colony PCR and Sanger sequencing. Primers and plasmids used in this study are listed in Tables 1 and 2, respectively. Plasmid constructs and further information are described in the Supporting Information S2.

## Results

### Design of the essential flavonoid synthetic pathway

Previous studies have demonstrated that efficient conversion of L-tyrosine to (2S)-naringenin or resveratrol is the limiting factor for de novo synthesis of (2S)-naringenin or resveratrol [11], [19]. In order to alleviate this bottleneck, firstly, the entire metabolic space for engineering the flavonoid pathway from L-tyrosine to (2S)-naringenin was exhaustively explored. There are no naturally occurring biosynthetic pathways for converting L-tyrosine into (2S)-naringenin in *E. coli*. Hence, selecting appropriate genetic sources for the enzymes in a pathway remains a challenging task.

As the first step of this pathway, TAL was chosen from the red yeast *Rhodotorula glutinis*, since this enzyme had been previously shown to have the highest *in vitro* enzyme activity toward L-tyrosine [11], [20], [21]. The enzymes used for conversion of *p*-coumaric acid to (2S)-naringenin, including 4CL from *Petroselinum crispum* (Pc4CL), CHS from *Petunia X hybrida*, and CHI from *Medicago sativa*, were chosen because these enzymes had been successfully utilized in previous studies [11], [19], [22]. To increase the supply of malonyl-CoA, which is a bottleneck of the native metabolism of *E. coli*, a recombinant malonate assimilation pathway from *Rhizobium trifolii* (*matB* and *matC*) was utilized [9]. All of the enzymes used in this study were codon-optimized for *E. coli* expression and synthesized in order to improve the expression of these enzymes.

### Assembling the essential synthetic pathway into three modules

The initial synthetic pathway was divided into three modules for two reasons. First, a previous study demonstrated that the low turnover number of TAL was partially due to inhibition caused by the buildup of coumaroyl-CoA [11]; hence the flavonoid pathway was divided at the intermediate coumaroyl-CoA to investigate whether this bottleneck could be alleviated by overexpressing the downstream pathway. Second, *matB* and *matC* were placed in an individual module to modulate the amount of malonyl-CoA, which is the bottleneck when overproducing flavonoids in *E. coli*. Therefore, this synthetic pathway was partitioned into three modules: (1) module one consisted of genes coding for TAL and 4CL; (2) module two consisted of genes coding for CHS and CHI; (3) module three consisted of genes *matB* and *matC* to modulate the amount of malonyl-CoA (Fig. 1).

Total modular expression was calculated using promoter strengths and gene copy numbers. The strengths of *T7* and *Trc* promoters were calculated as *T7* = 5, *Trc* = 1. The gene copy numbers of pRSFDuet-1 (RSF origin), pETDuet-1 (pBR322 origin), pCDFDuet-1 (CDF origin), and pACYCDuet-1 (p15A origin) were assigned from the published copy numbers for the origin of replication, which are 10, 20, 40, and 100, respectively [4], [12].

### Improving (2S)-naringenin production by modular pathway optimization

On the basis of previous studies [4], [11], [23], two design principles were developed to rationally design these three modules. One principle was that a high copy number plasmid would have negative effects on cell physiology when expressing heterologous pathways. Hence, the medium or low gene copy number plasmids, pETDuet-1 (pBR322 origin), pCDFDuet-1 (CDF origin), and pACYCDuet-1 (p15A origin), were used to modulate modular expression. The other was that module two should be overexpressed relative to module one to alleviate the inhibition of TAL caused by the buildup of coumaroyl-CoA.

**Figure 2:**
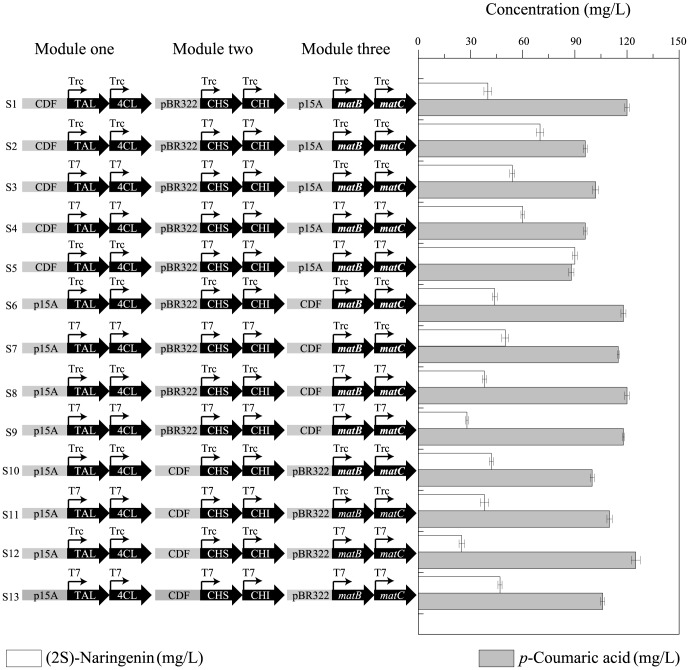
Optimization of (2S)-naringenin production from L-tyrosine by engineering three modules. pBR322: origin of pETDuet-1; CDF: origin of pCDFDuet-1; p15A: origin of pACYCDuet-1; T7: *T7* promoter; Trc: *Trc* promoter. S1–S13 denotes strains 1–13 constructed in this study. Gray bars: *p*-coumaric acid (mg/L); white bars: (2S)-naringenin (mg/L).

**Figure 3:**
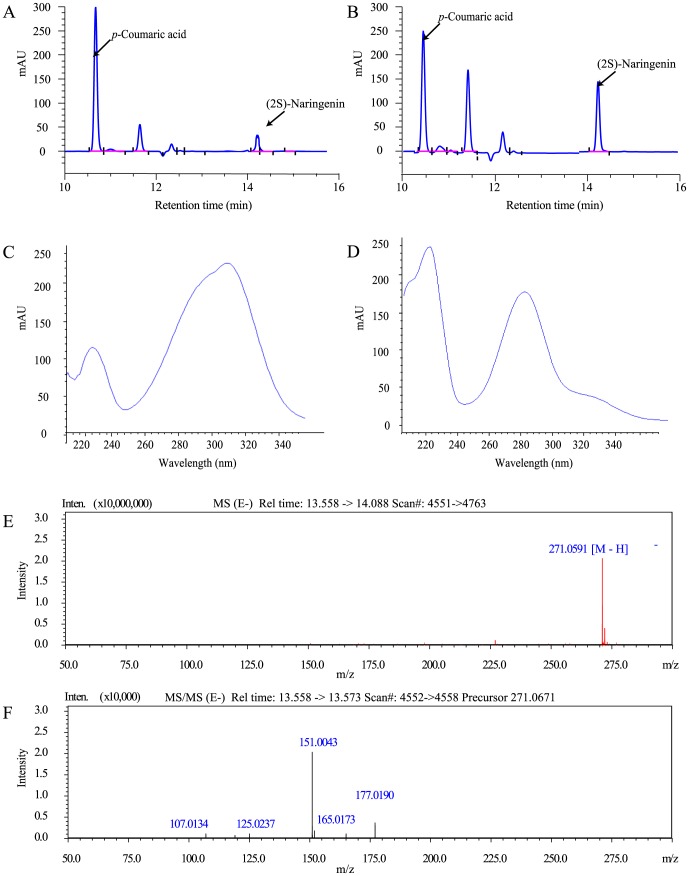
HPLC and LC-MS analysis of (2S)-naringenin and *p*-coumaric acid produced by engineered *E. coli* strains. A–B: Partial HPLC chromatograms show engineered strains have a significantly increased titer of (2S)-naringenin and a dramatically decreased titer of *p-*coumaric acid compared to the initial strain. A: Partial HPLC chromatograms of the initial strain; B: partial HPLC chromatograms of the optimized strain constructed through module engineering. C–D: HPLC chromatograms of *p*-coumaric acid (C) and (2S)-naringenin (D) in our sample. E–F: Selected ion chromatograms of (2S)-naringenin (*m/z* 271.0587 [M–H]^−^) produced by *E. coli*. E: LC/ESI-MS chromatogram of our compound; F: MS/MS spectrum of our compound.

**Figure 4:**
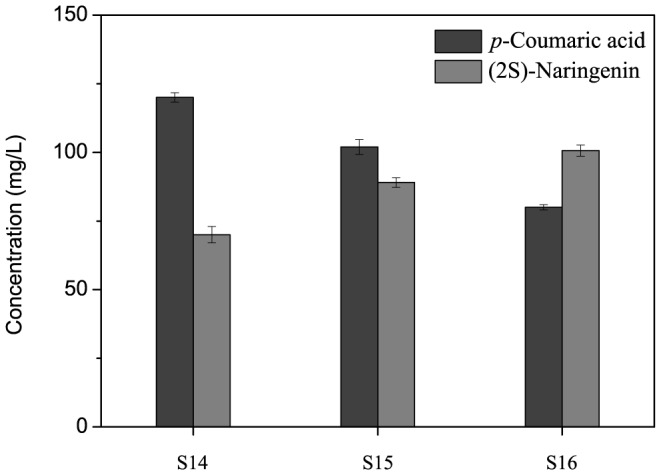
Assembling individual modules to enable de novo synthesis of (2S)-naringenin. S14–S16 denotes strains 14–16 constructed in this study. Gray bars: (2S)-naringenin (mg/L); dark gray bars: *p-*coumaric acid (mg/L). S14 means the new modular pathway from glucose to L-tyrosine was expressed at the plasmid of pRSFDuet-1; S15 means the new modular pathway from glucose to L-tyrosine was expressed at the plasmid of pCOLADuet-1; S16 means the new modular pathway from D-glucose to L-tyrosine was integrated into the *lacZ* locus of *E. coli* BL21 under *T7* promoter.

In order to alleviate the low turnover numbers of TAL, module two was overexpressed relative to module one (Fig. 2). In the first round of modular pathway optimization (S1–S3), module three was expressed at a constant value with the lowest gene copy number and a weaker promoter (p15A × *Trc*), while the metabolic space between modules one and two was varied. It was found that an appropriate metabolic space between modules one and two resulted in better yields. Similarly, in the second (S4–S5), third (S6–S7), fourth (S8–S9), fifth (S10–S11), and sixth (S12–S13) rounds, module three was expressed at constant values with different gene copy numbers and promoters, while the metabolic space between modules one and two was exhaustively explored. Finally, it was found that an appropriate metabolic space between modules one and two with special modular expression of module three (p15A × *T7*) would result in the best (2S)-naringenin production (Fig. 3). Once this metabolic balance was achieved, such strains were capable of producing 90.59 mg/L (2S)-naringenin from L-tyrosine (Fig. 4).

### Assembling individual modules to enable de novo synthesis of (2S)-naringenin

When the major pathway bottlenecks from L-tyrosine to (2S)-naringenin were alleviated, the next step was assembling a modular pathway from D-glucose to L-tyrosine to precede the identified optimal modules to enable de novo synthesis of (2S)-naringenin. To obtain (2S)-naringenin from D-glucose, strains exhibiting an enhanced capacity for L-tyrosine synthesis need to be constructed. In *E. coli*, the first rate-limiting step in the synthesis of L-tyrosine is the condensation of phosphoenolpyruvate (PEP) and erythrose 4-phosphate (E4P) catalyzed by DAHP synthase, which possesses three isoforms (encoded by *aroH*, *aroF*, and *aroG*). The second rate-limiting step in the L-tyrosine biosynthesis is found at the chorismate branch point with chorismate mutase/prephenate dehydrogenase (CM/PDH, *tyrA*) [24]. Based on known properties of the aromatic amino acid pathway, the feedback resistant derivatives of 3-deoxy-D-arabinoheptulosonate-7-phosphate (DAHP) synthase (*aroG* ^fbr^) [17] and chorismate mutase/prephenate dehydrogenase (*tyrA* ^fbr^) [16] were overexpressed to increase flux toward L-tyrosine. Hence, a new individual module consisting of *tyrA* ^fbr^ and *aroG* ^fbr^ was assembled with the identified optimal modules to enable de novo synthesis of (2S)-naringenin.

Two different plasmids, one with a high gene copy number (100) and the other with a low gene copy number (10), were modulated to rebalance the entire pathway. By expressing this module on the plasmid pRSFDuet-1 (highest gene copy number) or pCOLADuet-1 (lowest gene copy number), it was observed that the titer of the product (2S)-naringenin increased when the expression of this module decreased from the highest level to the lowest level (S14–S15). Our engineering methods require the use of antibiotic cassettes and plasmid-based expression, while marker and origin of replication incompatibilities can oftentimes arise between hosts and tools. Besides, the antibiotic cassettes and plasmid-based expression would result in an increase in the metabolic burden of host cells. Therefore, this new module was integrated into the *lacZ* locus of *E. coli* BL21 under the *T7* promoter. This resulted in the highest production titer of 100.64 mg/L (S16) directly from D-glucose (Fig. 4). It was found that the use of tyrosine-fermenting strains obviated the need to provide amino acids to the culture and elevated the yields of the final product.

Previous studies demonstrated that naringein chalcone could be converted to (2S)-naringenin or (2R)-naringenin spontaneously without CHI by raising the pH of the culture broth [7], [25]. Therefore, a combination of the three plasmids without the introduction of CHI, *i. e*., pCDF-Trc-TAL-Trc-4CL, pET-CHS, and pACYC-matC-matB, was transformed into BL21 (DE3) strain integrated with *tyrA* ^fbr^ and *aroG* ^fbr^. No (2S)-naringenin could be detected under the same culture conditions and analytical conditions as mentioned above.

## Discussion

The biosynthesis of flavonoids from safe, inexpensive, and renewable substrates is increasingly attracting attention due to concerns about food safety and environmetal issues [11], [26]. Previous studies have succeeded in producing (2S)-naringenin from *p*-coumaric acid [8], [9]. However, its high cost and poor water solubility restricted the direct application of phenylpropanoid acid precursors to industrial scale applications [23]. In particular, these precursors are unfavorable commercially in terms of food safety issues because most of the chemicals are obtained by chemical synthesis routes through acetylsalicyloyl chloride from the petroleum industry. In this study, a bacterial platform for (2S)-naringenin production directly from D-glucose was constructed. The strategy described here would decrease substrate-related costs and facilitate the extensive application of (2S)-naringenin in both the pharmaceutical and nutraceutical industries.

Previous studies have demonstrated the feasibility of de novo production of (2S)-naringenin [11]. The engineered strain could produce 46 mg/L (2S)-naringenin from D-glucose and up to 84 mg/L with the addition of the expensive fatty acid enzyme inhibitor, cerulenin [11]. However, the expressions of genes were only examined individually as part of the overall pathway, which would constrain production of the desired compound due to imbalance in the overall pathway [4], [12], [13]. In this study, the overall pathway, including the upstream pathway from D-glucose to L-tyrosine, the downstream pathway from L-tyrosine to (2S)-naringenin, and the malonate assimilation pathway, has been optimized. The optimal strain was capable of producing 100.64 mg/L (2S)-naringenin without the addition of cerulenin, which is the highest reported production titer from D-glucose in *E. coli*. This proves the necessity of varying the expressions of modules simultaneously.

In our previous work, for de novo production of (2S)-pinocembrin, the overall pathway from D-glucose to (2S)-pinocembrin was divided into four modules and expressions of the modules were varied simultaneously by modifying plasmid gene copy numbers [4]. However, it has become clear from previous studies that efficient conversion of aromatic amino acids to flavonoids is the limiting factor for de novo synthesis of these compounds [4], [11]. Therefore, the regulation of the overall pathway was divided into two separate steps to alleviate this bottleneck in this work. Furthermore, in order to find the best combination, both the plasmid gene copy number and promoter strength were varied to tune the modular expression. Finally, the production titer was increased 2.4-fold over that achieved in the previous study on (2S)-pinocembrin [4].

The engineered strains exhibited a significantly increased titer of (2S)-naringenin and a decreased titer of *p*-coumaric acid compared to the initial strain. However, *p*-coumaric acid accumulation was still observed in these strains. Further studies are required to achieve more efficient conversion of *p*-coumaric acid to (2S)-naringenin. In some particularly relevant studies, previous researchers have found that simultaneous deletion of genes *sdhA*, *adhE*, *brnQ*, and *citE* and overexpression of acetyl-CoA synthase, acetyl-CoA carboxylase, biotin ligase, and pantothenate kinase [10] or deletion of *fumC* and *sucC* and overexpression of ACC, PGK, GAPD, and PDH [8] could increase the (2S)-naringenin level dramatically. It is proposed that similar gains can be achieved in the strains described here to further enhance (2S)-naringenin production and decrease the accumulation of intermediates. A rational combination of these strategies could further efficiently close the gap between the current laboratory scale results and industrial scale production of flavonoids.

## Supporting Information

Supporting Information S1 DNA sequences of optimized genes.
(DOC)

Supporting Information S2 Description of plasmid constructs and further information.
(DOC)
