# Supplementary material for: Modular Optimization of Heterologous Pathways for De Novo Synthesis of (2S)-Naringenin in Escherichia coli
Source: PLoS One. 2014 Jul 2;9(7):e101492. doi: 10.1371/journal.pone.0101492 (PMC4079502; doi:10.1371/journal.pone.0101492)
Supplement: Supporting Information S1 — DNA sequences of optimized genes. (DOC) [file pone.0101492.s001.doc]

**Supporting Information S1. DNA sequences of optimized genes.**

***Rhodotorula glutinis* tyrosine ammonia lyase (TAL)** atggcgccgcgcccgacttctcaaagccaggcccgcacttgcccgaccacccaggttacccaagttgatatcgttgagaaaatgctggcggctccgactgatagcaccctggagctggacggttatagcctgaacctgggtgatgttgtgagcgctgcgcgtaagggtcgtccggttcgtgttaaagatagcgatgaaatccgcagcaaaatcgacaagagcgttgaatttctgcgcagccaactgagcatgtctgtttacggtgtgaccaccggctttggcggctccgcggacacccgcaccgaggacgcaattagcctgcaaaaggcgctgctggaacaccagctgtgtggtgtgctgccgagcagcttcgacagctttcgcctgggtcgtggtctggagaacagcctgccgctggaagttgttcgcggtgcaatgaccattcgtgtgaactctctgacccgtggccatagcgctgttcgtctggttgttctggaagcactgaccaactttctgaaccacggtattaccccgattgttccgctgcgcggtaccatctccgcgagcggcgatctgtctccactgtcttacattgcagcggcgattagcggtcacccggatagcaaagttcacgtggttcatgaaggcaaagagaagatcctgtacgcgcgcgaagcgatggcgctgtttaacctggagccggtggttctgggtccgaaggagggcctgggtctggtgaacggtaccgcagtttccgcgagcatggcaaccctggcactgcacgacgcgcacatgctgagcctgctgagccaatctctgaccgcgatgaccgtggaggcgatggttggtcacgcgggcagcttccatccattcctgcacgatgttacccgtccgcacccgacccaaatcgaggttgcgggtaacattcgcaaactgctggagggctctcgcttcgcggttcaccacgaggaagaggttaaggttaaggatgatgaaggcattctgcgtcaggatcgttatccgctgcgcaccagcccgcaatggctgggtccgctggtgtccgacctgattcacgctcatgccgttctgaccatcgaagcgggtcaaagcaccaccgataacccactgatcgatgttgagaacaagaccagccatcacggtggcaactttcaagcggcagcggttgccaacactatggaaaagacccgtctgggcctggcccaaatcggtaaactgaacttcacccagctgaccgagatgctgaacgcgggcatgaaccgtggcctgccgagctgcctggcggctgaagacccatccctgagctatcattgcaaaggtctggacattgcggcggctgcatataccagcgaactgggccacctggctaacccggttaccacccacgttcaaccggctgaaatggcaaaccaggcggtgaacagcctggcgctgattagcgcacgtcgtaccaccgaatctaacgacgttctgtccctgctgctggcaacccacctgtactgcgtgctgcaggcgatcgacctgcgtgcgattgagttcgagttcaagaaacagtttggtccggccattgttagcctgatcgaccaacactttggtagcgcgatgaccggtagcaacctgcgtgatgagctggttgaaaaggttaacaagactctggccaagcgtctggagcaaaccaacagctacgatctggttccgcgctggcacgacgcttttagcttcgctgcaggcactgttgttgaggttctgtccagcaccagcctgagcctggcggccgtgaacgcatggaaggttgcggcagccgagagcgcgatctccctgacccgccaggttcgtgaaaccttttggtccgctgcaagcacctccagcccggcgctgtcttacctgagcccgcgcacccagatcctgtacgcatttgtgcgtgaggaactgggtgttaaagcccgccgtggtgacgttttcctgggtaaacaagaagttaccatcggcagcaacgttagcaagatttacgaagccatcaagagcggccgtatcaacaacgttctgctgaagatgctggcataa

***Petroselinum crispum* 4-coumarate:CoA ligase (4CL)**

atgggtgactgcgttgccccgaaagaggatctgatcttccgcagcaaactgccggacatttacattccaaagcatctgccgctgcatacctattgttttgagaacatcagcaaggttggcgacaagagctgtctgatcaacggcgcaaccggcgaaacctttacctacagccaggttgagctgctgtcccgtaaagttgccagcggcctgaacaagctgggcattcaacaaggtgataccattatgctgctgctgccgaactccccggagtactttttcgctttcctgggtgcgagctatcgcggtgcaatcagcactatggcgaacccattctttaccagcgcagaagtgatcaagcaactgaaagcgagccaagcgaagctgattatcacccaggcatgctatgttgacaaggttaaggactacgcagcggagaaaaacatccagatcatttgtattgacgatgcaccgcaggattgcctgcactttagcaagctgatggaagcggatgagagcgaaatgccggaagtggttattaacagcgatgatgtggtggcactgccgtacagctctggcaccaccggcctgccgaaaggcgttatgctgacccacaagggtctggttaccagcgttgcacaacaggtggatggtgataacccgaacctgtatatgcactccgaggatgttatgatctgcatcctgccactgttccatatctatagcctgaacgctgttctgtgttgtggtctgcgtgcgggcgttaccattctgatcatgcaaaagttcgacattgtgccgtttctggagctgattcagaagtataaggttaccattggtccgtttgttccgccgatcgtgctggccatcgcgaaaagcccggttgttgacaagtacgacctgtctagcgtgcgcaccgttatgagcggtgcagcgccgctgggtaaagagctggaggacgctgttcgtgcgaaattcccgaacgcgaagctgggtcaaggctatggcatgaccgaagccggtccggttctggcgatgtgtctggcgttcgccaaagagccgtatgagattaagtctggcgcatgcggtaccgttgtgcgtaacgccgagatgaaaatcgttgacccagaaaccaacgcgtctctgccgcgtaaccagcgtggtgagatttgcatccgtggtgatcagattatgaaaggttacctgaacgacccggaaagcacccgcaccaccatcgacgaagagggttggctgcacaccggtgacattggtttcatcgacgatgacgatgaactgttcattgttgatcgtctgaaagaaatcattaagtacaaaggttttcaagttgctccggcggagctggaagcactgctgctgacccacccgaccatcagcgatgccgcggtggttccgatgattgacgagaaagcgggtgaagtgccagtggcgtttgttgtgcgtaccaacggttttaccaccaccgaagaagaaatcaaacaatttgtgagcaaacaggttgtgttctacaaacgtatcttccgcgttttcttcgttgacgctattccgaaatccccgagcggcaagattctgcgtaaggatctgcgcgctcgtattgcgagcggcgacctgccgaagtaa

***Petunia X hybrida* Chalcone synthase (CHS)**

ATGGTTACGGTGGAAGAATACCGCAAAGCTCAACGCGCTGAAGGCCCGGCGACGGTGATGGCGATTGGCACGGCAACCCCGACGAACTGTGTTGATCAGAGCACCTATCCGGACTATTACTTTCGTATCACCAACTCTGAACATAAAACGGATCTGAAAGAAAAATTCAAACGTATGTGCGAAAAAAGCATGATCAAAAAACGCTATATGCACCTGACCGAAGAAATTCTGAAAGAAAATCCGAGCATGTGTGAATACATGGCACCGTCTCTGGATGCTCGCCAGGACATTGTGGTTGTCGAAGTGCCGAAACTGGGTAAAGAAGCGGCCCAGAAAGCGATCAAAGAATGGGGCCAACCGAAATCAAAAATTACCCATCTGGTCTTTTGCACCACGTCGGGTGTGGATATGCCGGGTTGTGACTATCAACTGACGAAACTGCTGGGTCTGCGTCCGAGCGTGAAACGCCTGATGATGTACCAGCAAGGCTGCTTCGCAGGCGGTACCGTTCTGCGTCTGGCGAAAGATCTGGCCGAAAACAATAAAGGTGCGCGTGTTCTGGTGGTGTGTAGTGAAATCACCGCTGTTACGTTTCGTGGTCCGAACGATACGCACCTGGACTCCCTGGTTGGCCAGGCCCTGTTCGGTGATGGTGCAGGTGCCATTATCATTGGTAGCGACCCGATTCCGGGCGTTGAACGTCCGCTGTTTGAACTGGTCAGCGCAGCTCAAACCCTGCTGCCGGATAGCCACGGCGCAATTGACGGTCACCTGCGTGAAGTCGGTCTGACGTTCCATCTGCTGAAAGATGTGCCGGGCCTGATCTCAAAAAACATTGAAAAAAGCCTGGAAGAAGCGTTTCGCCCGCTGAGTATCTCCGATTGGAACAGCCTGTTCTGGATTGCACATCCGGGCGGCCCGGCAATCCTGGACCAGGTCGAAATTAAACTGGGTCTGAAACCGGAAAAACTGAAAGCGACCCGTAATGTTCTGTCAAACTACGGCAATATGAGCTCTGCCTGCGTCCTGTTTATTCTGGATGAAATGCGCAAAGCATCGGCTAAAGAAGGTCTGGGCACCACGGGTGAAGGCCTGGAATGGGGCGTGCTGTTCGGCTTTGGTCCGGGTCTGACGGTGGAAACGGTGGTTCTGCATAGTGTGGCTACCTAA

***Medicago sativa* chalcone isomerase (CHI)**

ATGGCAGCAAGCATTACGGCAATCACGGTTGAAAATCTGGAATATCCGGCGGTCGTTACCTCTCCGGTCACGGGCAAATCATACTTTCTGGGCGGTGCCGGTGAACGTGGTCTGACCATTGAGGGTAACTTTATCAAATTCACGGCAATTGGCGTTTATCTGGAAGATATCGCGGTCGCCTCACTGGCGGCCAAATGGAAAGGTAAAAGCTCTGAAGAACTGCTGGAAACCCTGGATTTTTACCGTGACATTATCTCAGGCCCGTTCGAAAAACTGATCCGTGGTTCGAAAATTCGCGAACTGAGCGGCCCGGAATATTCTCGCAAAGTCATGGAAAACTGCGTGGCTCATCTGAAATCCGTCGGCACGTACGGTGACGCAGAAGCTGAAGCGATGCAGAAATTTGCCGAAGCATTCAAACCGGTGAATTTTCCGCCGGGTGCCAGTGTTTTCTATCGTCAATCCCCGGATGGCATCCTGGGTCTGTCATTTTCGCCGGACACCAGCATCCCGGAAAAAGAAGCAGCTCTGATTGAAAATAAAGCTGTGAGTTCCGCGGTTCTGGAAACGATGATTGGCGAACACGCGGTTTCTCCGGATCTGAAACGCTGTCTGGCTGCTCGCCTGCCGGCTCTGCTGAATGAAGGTGCCTTTAAAATCGGTAACTGA

***Rhizobium trifolii* malonate synthetase (*matB*)**

ATGGTTAGCAACCACCTGTTTGATGCGATGCGTGCGGCAGCACCGGGCAACGCTCCGTTCATTCGTATTGACAACACCCGTACCTGGACCTACGATGACGCGTTTGCCCTGAGTGGTCGTATTGCATCCGCTATGGATGCACTGGGCATCCGTCCGGGTGACCGTGTCGCCGTGCAGGTTGAAAAAAGTGCGGAAGCCCTGATTCTGTATCTGGCGTGCCTGCGTTCCGGTGCAGTGTATCTGCCGCTGAACACCGCGTACACGCTGGCCGAACTGGATTATTTCATCGGCGACGCCGAACCGCGTCTGGTGGTTGTCGCAAGCTCTGCACGCGCTGGTGTTGAAACGATTGCAAAACCGCGTGGCGCTATCGTCGAAACCCTGGATGCAGCAGGTAGTGGTTCCCTGCTGGATCTGGCGCGTGACGAACCGGCCGATTTTGTGGACGCATCACGCTCGGCTGATGACCTGGCAGCTATTCTGTACACGAGCGGCACCACGGGTCGTTCTAAAGGCGCAATGCTGACCCACGGCAACCTGCTGAGCAATGCGCTGACGCTGCGTGATTTCTGGCGCGTTACCGCAGGCGACCGCCTGATTCACGCTCTGCCGATCTTTCATACCCACGGTCTGTTCGTGGCGACCAATGTTACGCTGCTGGCGGGCGCCTCAATGTTTCTGCTGTCGAAATTCGATCCGGAAGAAATTCTGTCACTGATGCCGCAGGCCACGATGCTGATGGGTGTCCCGACCTTTTATGTGCGTCTGCTGCAGTCGCCGCGCCTGGATAAACAAGCAGTGGCTAACATTCGCCTGTTTATCAGCGGTTCTGCGCCGCTGCTGGCAGAAACCCATACGGAATTTCAAGCACGTACCGGTCACGCTATTCTGGAACGCTACGGCATGACCGAAACGAACATGAATACGAGTAATCCGTATGAAGGTAAACGTATCGCAGGCACCGTGGGTTTTCCGCTGCCGGATGTCACCGTGCGTGTTACGGACCCGGCAACCGGTCTGGCTCTGCCGCCGGAACAGACCGGCATGATTGAAATCAAAGGTCCGAACGTTTTTAAAGGCTACTGGCGTATGCCGGAAAAAACGGCGGCCGAATTTACCGCGGATGGCTTTTTCATTTCTGGCGACCTGGGTAAAATCGATCGTGACGGCTATGTCCATATTGTGGGCCGCGGTAAAGATCTGGTGATTAGCGGCGGTTATAATATCTACCCGAAAGAAGTTGAAGGTGAAATTGATCAAATCGAAGGCGTGGTTGAATCTGCGGTTATTGGTGTCCCGCACCCGGATTTTGGCGAAGGTGTCACCGCAGTCGTGGTTCGTAAACCGGGTGCAGCTCTGGATGAAAAAGCGATCGTTTCCGCACTGCAGGACCGTCTGGCACGCTATAAACAACCGAAACGTATTATCTTCGCAGAAGATCTGCCGCGCAACACGATGGGCAAAGTCCAGAAAAACATCCTGCGCCAACAATACGCCGACCTGTACACCCGCACCTGA

***Rhizobium trifolii* malonate carrier protein (*matC*)**

ATGGGCATCGAACTGCTGAGTATTGGTCTGCTGATTGCTATGTTTATTATTGCTACGATTCAACCGATTAACATGGGTGCTCTGGCATTCGCAGGCGCTTTTGTGCTGGGTAGCATGATTATCGGCATGAAAACCAACGAAATTTTCGCAGGCTTTCCGTCTGACCTGTTTCTGACCCTGGTGGCGGTTACGTACCTGTTTGCGATTGCCCAGATCAATGGCACCATCGACTGGCTGGTTGAATGCGCGGTGCGTCTGGTTCGTGGCCGCATTGGTCTGATCCCGTGGGTGATGTTCCTGGTTGCGGCCATTATCACCGGTTTTGGTGCACTGGGTCCGGCAGCTGTTGCAATTCTGGCACCGGTCGCACTGAGCTTCGCAGTGCAATATCGCATTCATCCGGTTATGATGGGTCTGATGGTCATCCACGGCGCACAGGCTGGCGGTTTTTCACCGATTTCGATCTACGGCGGTATTACCAACCAAATCGTGGCAAAAGCAGGTCTGCCGTTCGCACCGACGAGTCTGTTTCTGAGCAGCTTTTTCTTTAATCTGGCAATTGCTGTCCTGGTGTTCTTTGTGTTTGGCGGTGCACGTGTTATGAAACACGATCCGGCTTCTCTGGGTCCGCTGCCGGAACTGCATCCGGAAGGCGTGAGCGCGTCTATTCGTGGTCATGGCGGCACCCCGGCAAAACCGATCCGCGAACATGCGTATGGCACCGCAGCAGACACGGCAACCACGCTGCGTCTGAACAATGAACGCATTACCACGCTGATCGGTCTGACCGCACTGGGTATTGGTGCACTGGTTTTCAAATTTAACGTCGGTCTGGTGGCTATGACCGTGGCAGTGGTTCTGGCACTGCTGAGCCCGAAAACGCAGAAAGCAGCTATTGATAAAGTCAGTTGGTCCACCGTGCTGCTGATCGCGGGTATTATCACGTATGTTGGCGTCATGGAAAAAGCGGGCACCGTTGACTACGTCGCCAATGGTATTAGTTCCCTGGGTATGCCGCTGCTGGTCGCGCTGCTGCTGTGTTTCACCGGCGCCATCGTGTCCGCGTTTGCCTCATCGACGGCACTGCTGGGTGCTATTATCCCGCTGGCCGTTCCGTTCCTGCTGCAGGGCCATATTAGTGCAATCGGTGTCGTGGCGGCCATTGCTATCTCCACCACGATTGTGGATACCAGCCCGTTTTCTACGAACGGCGCGCTGGTTGTCGCAAATGCTCCGGATGACTCACGTGAACAGGTTCTGCGCCAACTGCTGATCTATTCGGCCCTGATTGCTATTATTGGTCCGATTGTCGCCTGGCTGGTTTTCGTTGTGCCGGGTCTGGTCTAA
