# Supplementary material for: Modular Optimization of Heterologous Pathways for De Novo Synthesis of (2S)-Naringenin in Escherichia coli
Source: PLoS One. 2014 Jul 2;9(7):e101492. doi: 10.1371/journal.pone.0101492 (PMC4079502; doi:10.1371/journal.pone.0101492)
Supplement: Supporting Information S2 — Description of plasmid constructs and further information. (DOC) [file pone.0101492.s002.doc]

**Supporting Information S2. Description of plasmid constructs and further information.**

Plasmid constructs and further information are described below.

pACYC-TAL was constructed by digesting TAL from pUC57-TAL (synthesized by GeneScript, Nanjing, China) into the *Nco*I/*Hin*dIII sites of pACYCDuet-1. pACYC-TAL-4CL was constructed by digesting 4CL from pUC57-4CL (synthesized by GeneScript, Nanjing, China) into the *Nde*I/*Bln*I sites of pACYC-TAL. The TAL-4CL fragment was digested from pACYC-TAL-4CL by *Nco*I and *Bln*I and independently inserted to the *Nco*I/*Bln*I site of pCDFDuet-1 resulted in pCDF-TAL-4CL. Primers Pf_Trc(*Fse*I) and Pr_Trc(*Eco*NI) were used to clone the *Trc* promoter, multi-cloning sites and *rrnB* terminator from pTrcHis2B to the sites of *Eco*NI/*Fse*I in pCDFDuet-1, pETDuet-1 and pACYCDuet-1, resulted in pCDFD-Trc, pET-Trc and pACYC-Trc. TAL were independently cloned into pCDFD-Trc and pACYC-Trc using primers Pf_TAL(*Nco*I) and Pr_TAL(*Eco*RI) with the restriction enzymes *Nco*I and *Eco*RI, respectively. This resulted in pCDF-Trc-TAL and pACYC-Trc-TAL. 4CL was independently cloned into pCDFD-Trc and pACYC-Trc using primers Pf_4CL(*Nco*I) and Pr_4CL(*Hin*dIII) with the restriction enzymes *Nco*I and *Hin*dIII, resulted in pCDF-Trc-4CL and pACYC-Trc-4CL. The pTrc-4CL region including *Trc* promoter and 4CL was amplified with primers Pf_Ptrc4CL(*Eco*RI) and Pr_4CL(*Hin*dIII) and cloned into pCDF-Trc-TAL and pACYC-Trc-TAL with *Eco*RI and *Hin*dIII to construct pCDF-Trc-TAL-Trc-4CL and pACYC-Trc-TAL-Trc-4CL.

PET-CHS was constructed by cloning CHS from pUC57-CHS (synthesized by GeneScript, Nanjing, China) into the *Nco*I/*Hin*dIII sites of pETDuet-1. pET-CHS-CHI was constructed by cloning CHI from pUC57-CHI (synthesized by GeneScript, Nanjing, China) into the *Nde*I/*Bln*I sites of pET-CHS. The CHS-CHI fragment was digested from pET-CHS-CHI by *Nco*I and *Bln*I and independently inserted to the *Nco*I/*Bln*I site of pCDFDuet-1 resulted in pCDF-CHS-CHI. CHS was independently cloned into pCDFD-Trc and pETD-Trc using primers Pf_CHS(*Nco*I) and Pr_CHS(*Eco*RI) with the restriction enzymes *Nco*I and *Eco*RI, respectively. This resulted in pCDF-Trc-CHS and pETD-Trc-CHS. CHI was independently cloned into pCDFD-Trc and pETD-Trc using primers Pf_CHI(*Nco*I) and Pr_CHI(*Hin*dIII) with the restriction enzymes *Nco*I and *Hin*dIII, resulted in pCDF-Trc-CHI and pETD-Trc-CHI. The pTrc-CHI region including *Trc* promoter and CHI was amplified with primers Pf_PtrcCHI(*Eco*RI) and Pr_CHI(*Hin*dIII) and cloned into pCDF-Trc-CHS and pETD-Trc-CHS with *Eco*RI and *Hin*dIII to construct pCDF-Trc-CHS-Trc-CHI and pET-Trc-CHS-Trc-CHI.

*matB* was cloned into pACYCDuet-1 from pUC57-matB (synthesized by GeneScript, Nanjing, China) in between the *Nde*I and *Kpn*I sites. *matC* (synthesized by GeneScript, Nanjing, China) was cloned into the resulting product in between the *Eco*RI and *Hin*dIII sites. The resulting plasmid was named pACYC-matC-matB. The *matC*-*matB* fragment was digested from pACYC-matC-matB by *Eco*RI and *Kpn*I and independently inserted to the *Eco*RI/*Kpn*I sites of pCDFDuet-1 and pETDuet-1. This resulted in the plasmids of pCDF-matC-matB and pET-matC-matB. The *matB* was first independently cloned into pCDFD-Trc, pETD-Trc and pACYC-Trc using primers Pf_matB(*Nco*I) and Pr_matB(*Hin*dIII) from pET-matC-matB with the restriction enzymes *Nco*I and *Hin*dIII. This resulted in pCDF-Trc-matB, pET-Trc-matB and pACYC-Trc-matB. *matC* was first independently cloned into pCDFD-Trc, pETD-Trc and pACYC-Trc using primers Pf_matC(*Nco*I) and Pr_matC(*Eco*RI) from pET-matC-matB with the restriction enzymes *Nco*I and *Eco*RI. This resulted in pCDF-Trc-matC, pET-Trc-matC, pACYC-Trc-matC. The pTrc-matB region including *Trc* promoter and the gene of *matB* was amplified from pCDF-Trc-matB with primers Pf_PtrcmatB(*Eco*RI) and Pr_matB(*Hin*dIII) and cloned into pCDF-Trc-matC, pET-Trc-matC and pACYC-Trc-matC with the enzyme sites *Eco*RI and *Hin*dIII to construct pCDF-Trc-matC-Trc-matB, pET-Trc-matC-Trc-matB and pACYC-Trc-matC-Trc-matB.

pRSF-aroG^fbr^-tyrA^fbr^ was constructed through the digestion of pCDF- aroG^fbr^-tyrA^fbr^ and pRSFDuet-1 with *Nco*I and *Bln*I, followed by ligation of the appropriate fragments. pCOLA-aroG^fbr^-tyrA^fbr^ was constructed through the digestion of pCDF-aroG^fbr^-tyrA^fbr^ and pCOLADuet-1 with *Nco*I and *Bln*I, followed by ligation of the appropriate fragments.
